# Randomized comparison of the i-gel™, the LMA Supreme™, and the Laryngeal Tube Suction-D using clinical and fibreoptic assessments in elective patients

**DOI:** 10.1186/1471-2253-12-18

**Published:** 2012-08-07

**Authors:** Sebastian G Russo, Stephan Cremer, Tamara Galli, Christoph Eich, Anselm Bräuer, Thomas A Crozier, Martin Bauer, Micha Strack

**Affiliations:** 1 Department of Anaesthesiology, Emergency and Intensive Care Medicine, University Medical Centre Göttingen, Robert-Koch-Straße 40, Göttingen, 37083, Germany; 2 Current affiliation: Department of Anaesthesia and Emergency Care, University Hospital San Giovanni Battista di Torino, Turin, Italy; 3 Current affiliation: Department of Anaesthesia, Paediatric Intensive Care and Emergency Medicine, Children’s Hospital Auf der Bult, Hannover, Germany; 4 Georg-Elias-Müller Institute for Psychology, Georg-August University, Göttingen, Germany

**Keywords:** Laryngeal mask airway, Leak pressure, Laryngeal Tube

## Abstract

**Background:**

The i-gel™, LMA-Supreme (LMA-S) and Laryngeal Tube Suction-D (LTS-D) are single-use supraglottic airway devices with an inbuilt drainage channel. We compared them with regard to their position *in situ* as well as to clinical performance data during elective surgery.

**Methods:**

Prospective, randomized, comparative study of three groups of 40 elective surgical patients each. Speed of insertion and success rates, leak pressures (LP) at different cuff pressures, dynamic airway compliance, and signs of postoperative airway morbidity were recorded. Fibreoptic evaluation was used to determine the devices’ position *in situ*.

**Results:**

Leak pressures were similar (i-gel™ 25.9, LMA-S 27.1, LTS-D 24.0 cmH_2_O; the latter two at 60 cmH_2_O cuff pressure) as were insertion times (i-gel™ 10, LMA-S 11, LTS-D 14 sec). LP of the LMA-S was higher than that of the LTS-D at lower cuff pressures (p <0.05). Insertion success rates differed significantly: i-gel™ 95%, LMA-S 95%, LTS-D 70% (*p* <0.05). The fibreoptically assessed position was more frequently suboptimal with the LTS-D but this was not associated with impaired ventilation. Dynamic airway compliance was highest with the i-gel™ and lowest with the LTS-D (*p <*0.05). Airway morbidity was more pronounced with the LTS-D (*p <*0.01).

**Conclusion:**

All devices were suitable for ventilating the patients’ lungs during elective surgery.

**Trial registration:**

German Clinical Trial Register DRKS00000760

## Background

Since the introduction of the classic laryngeal mask airway the field of supraglottic airway devices (SGA) has experienced a remarkable evolution and SGA are now routinely used in clinical anaesthesia. Newer SGA have an inbuilt drainage channel to facilitate the efflux of gastric fluid and gas and allow the insertion of a gastric tube.

Many SGA are available in single-use versions [1,2]. The i-gel™ (Intersurgical Ltd.), the LMA-Supreme™ (LMA-S; The Laryngeal Mask Company Ltd), and the Laryngeal Tube Suction - D (LTS-D, VBM Medical GmbH) are disposable SGA with inbuilt drainage channel. The i-gel™ has a non-inflatable, gel-filled cuff [3] while the LMA-S is a disposable, pre-curved modification of the older LMA-ProSeal™ [4]. The LTS-D is the disposable version of the double-cuffed laryngeal tube [5].

These three SGA have previously been evaluated alone or in pair-wise comparisons but differing study designs make it difficult to compare the results [2,6-8]. To the best of our knowledge, no previous study has compared the three devices in a single study setting. We therefore compared the three SGA in a, randomized, prospective clinical study with a detailed evaluation of their performance. We evaluated the leak pressures (LP) at different cuff volumes and cuff pressures, the speed of insertion, the insertion success rates, the fibreoptically determined positions, dynamic airway compliance and signs of airway morbidity.

## Methods

With the approval of our institutional ethics committee and after having obtained written informed consent, patients were recruited for this prospective, randomized clinical study (German Clinical Trial Register number DRKS00000760). Inclusion criteria were: age >18 years and scheduled elective surgical intervention in the supine position with predicted anaesthesia duration between 60 and 180 minutes. Exclusion criteria were: BMI >35 kg/m^2^, ASA status III or higher, known risk of aspiration, known or predicted difficult airway. The patients were assigned to their groups with a computer-generated randomisation list (http://www.randomizer.org). The sealed envelope method was used for blinding.

Leak pressure (LP) was the primary endpoint. Secondary endpoints were speed of insertion, insertion success rates, fibreoptically assessed *in situ* position, dynamic airway compliance and signs of airway morbidity.

### Anaesthesia

The patients were premedicated with midazolam (7.5 mg p.o.) thirty minutes prior to anaesthesia induction. Anaesthesia was induced with sufentanil (0.3-0.5 μg/kg) and propofol (1.5-2.5 mg/kg) and maintained during the measurement period with a propofol infusion (5-8 mg/kg/h) and repeated injections of sufentanil when necessary. No muscle relaxants or opioids other than sufentanil were given.

### Airway management and ventilation

Two senior anaesthesia registrars (SC and TG) skilled in placing SGA performed all cases. Although the recommended insertion techniques of the three SGA are quite similar the investigators were required to perform a minimum of 15 insertions with all three devices before starting patient recruitment, particularly because they had previous experience with reusable laryngeal mask type devices (classical laryngeal mask airway and PLMA) but not i-gel™ and LMA-S. Depth of anaesthesia was assessed by performing a jaw thrust manoeuvre [9,10]. The size of the device was determined according to the manufacturers’ weight-based recommendations.

If two attempts to insert the initially randomized SGA failed, the study protocol prescribed a change to one of the other two devices selected randomly (coin toss). If two attempts with the second SGA device were also unsuccessful the trachea was intubated.

Failed insertion of the SGA was defined as the inability to position the device within 60 seconds, an air leak through the drainage channel during positive pressure ventilation despite corrective manoeuvres (e.g. deeper insertion or up-and-down-manoeuvre) [11], inability to introduce a suction catheter (12Ch for the i-gel™, 16Ch for the LMA-S and LTS-D) beyond the tip of the device, inability to establish successful ventilation with a stable end-expiratory CO_2_ signal with a targeted expiratory tidal volume of 7 ml/kg because of leakage or airway obstruction.

The time required for successful insertion was defined as the time from placing the SGA in the front of the patient’s mouth to its placement in the correct position. A non-blinded observer who was not involved in the study recorded the time needed for insertion.

After successful insertion, the cuff of the LMA-S was inflated to a pressure of 60 cmH_2_O. The cuff of the LTS-D was initially inflated with the volume indicated on the syringe provided by the manufacturer for emergency use. The cuff pressure was measured at this inflation volume and air was then withdrawn until cuff pressure was also 60 cmH_2_O.

Ventilation was pressure-controlled (PCV) with a positive end-expiratory pressure (PEEP) of 3 cmH_2_O, a respiratory rate between 14 and 16 and an inspiratory to expiratory ratio of 1:1.5. It was adapted to give an end-tidal CO_2_ of 35-40 mmHg.

Leak pressure was determined as a function of cuff pressure for the SGA with inflatable cuffs (LMA-S and the LTS-D). Cuff pressure was measured with a manometer (VBM Medical GmbH, Sulz, Germany; range from 0 to 110 cmH_2_O) that was connected to the SGA through a three-way stopcock with an attached syringe. Air was injected until the pre-determined cuff pressure was obtained and the required inflation volume was recorded. Cuff pressures started at 0 cmH_2_0 (completely deflated and equilibrated to ambient pressure) and were increased in 10 cmH_2_0 increments to 60 cmH_2_0. It was further increased in the LTD-S in two 20 cmH_2_0 increments to a maximum of 100 cmH_2_O. The pressure limit of the anaesthesia circuit was set to 35 cmH_2_O and airway pressure was increased steadily with a continuous flow of oxygen (3 l/min). Leakage was defined as air escape audible with a stethoscope placed on the larynx, and leak pressure was defined as the airway pressure at which leakage was first detected [12]. After these measurements the cuff was inflated to 60 cmH_2_O and kept at that pressure for the remainder of the study duration.

Airway pressures (PAW) and tidal volumes (V_t_) were recorded and averaged over one minute after insertion, as well as after equilibration of ventilator settings. Dynamic airway compliance (C_dyn_) was calculated using the formula ${\text{C}}_{\text{dyn}}={\text{V}}_{\text{t}}/\left({\text{PAW}}_{\text{max}}-\text{PEEP}\right)$. The LP defined the maximum inspiratory airway pressure setting.

### Fibreoptic evaluation

Fibreoptic (FO) evaluation of the SGA’s position was performed after successful insertion and determination of the airway pressures and tidal volumes. The position was assessed using a previously described four points score (1 = only vocal cords seen; 2 = cords and/or arytenoids seen; 3 = only epiglottis seen; 4 = other (e.g. cuff, pharynx, etc)) [13].

If an unexpected reduction in tidal volume occurred during stable anaesthesia and unchanged ventilator settings, the airway was assessed with the fibrescope for dislocation of the device or obstruction due to glottic narrowing or laryngospasm. Glottic narrowing was differentiated from laryngospasm by a partial closure of the vocal cords that was not reversed by deepening anaesthesia or by the administration of a neuromuscular blocker.

### Airway morbidity

All devices were evaluated for traces of blood on the mask bowl (LMA-S, i-gel™) or the airway apertures and cuff (LTS-D). The patients were questioned about sore throat, discomfort during swallowing and hoarseness at one hour and at 24 hours after anaesthesia. These complaints were classified as none, mild, or severe.

### Statistical analysis

Published data on leak pressures were used to estimate the necessary sample size. Assuming a mean LP of 24 cmH_2_O for the i-gel™ [3,14], 26 cmH_2_O for the LMA-S [6,7,15], and 28 cmH_2_O for the LTS-D [8,16] and an assumed standard deviation of 5 cmH_2_O for all devices, a total sample size of 83 was calculated to detect differences with 90% power and a significance level of 0.05 [17]. To allow for potential dropouts, a sample size of 40 patients per group was chosen.

The data were documented in an Excel™ spreadsheet and analyzed using SPSS Statistics™ software (IBM SPSS Inc., Chicago, IL, USA). Depending on the level of measurement of the dependent variables, ANOVA, rank variance analysis (Kruskall-Wallis), or multinomial logistic regressions and chi-square tests were used.

## Results

**Figure 1:**
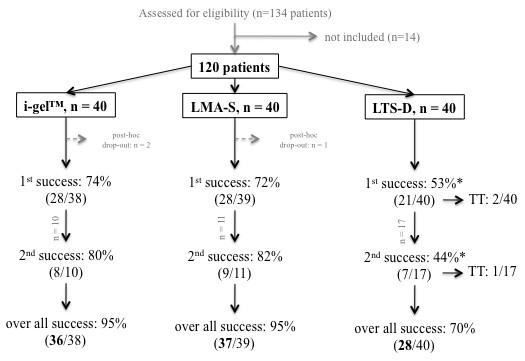
**Flow chart of the study, showing the subdivision into groups corresponding to each supraglottic airway device.** 1st or 2nd success = insertion success rate for the first or the second insertion attempt. i-gel™; LMA-S = LMA-Supreme™; LTS-D = Laryngeal Tube Suction-D; TT = tracheal tube.

One hundred and thirty-four patients on the surgical list were identified as possible participants. Of these, 14 were not eligible due to exclusion criteria. The remaining 120 patients fulfilled all criteria, and after having given written informed consent were recruited to participate in the study. They were randomly allocated to receive one of the three SGA. Three patients were excluded from further analysis because of protocol violation (wrong sized device; one LMA-S, two i-gel; Figure 1). The results of 117 patients, 78 female and 39 male, were analyzed. The groups did not differ with regard to duration of anaesthesia (120 ± 48 minutes, *p* = 0.63). For further biometric data see Table 1.

**Table 1:** Biometric data of the intent-to-treat groups

	**BMI**	**Height (cm)**	**Age (yrs)**	**Male / Female (n)**
**i-gel™**	25.9 ± 4.5	172 ± 11	48 ± 17	16 / 24
**LMS-S**	26.8 ± 4.3	172 ± 10	50 ± 17	13 / 27
**LTS-D**	25.8 ± 3.5	168 ± 10	51 ± 16	10 / 30
***p-*value**	= 0.508	= 0.168	= 0.641	= 0.272

LMA-S = LMA-Supreme™; LTS-D = Laryngeal Tube Suction-D.

### Leak pressure for the intention-to-treat patients

**Figure 2:**
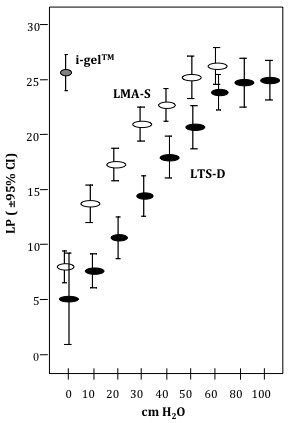
**Leak pressure (LP) versus cuff pressure shown for the i-gel™, the LMA-Supreme™ and the Laryngeal Tube Suction-D.** LP is given in cmH_2_0. The grey circle represents the i-gel™, white circles represent the LMA-S, black circles represent the LTS-D.

There was no significant difference in LP between the three devices (cuff pressure = 60 cmH_2_0 for LMA-S and LTS-D): i-gel™ 25.9 ± 5.6; LMA-S 27.1 ± 5.2; LTS-D 24.0 ± 3.9 (*p* = 0.184; see also Figure 2).

In both the LMA-S and the LTS-D, LP increased with increasing cuff pressure. Leak pressure was significantly higher for the LMA-S at lower cuff pressures (*p* <0.05, Figure 2). In the LTS-D, LP increased only insignificantly at cuff pressures above 60 cmH_2_0 (Figure 2).

Injected air volumes of 24 ± 5 ml and 58 ± 15 ml were required to generate a cuff pressure of 60 cmH_2_O in a size four LMA-S and LTS-D, respectively. Generating cuff pressures of 80 and 100 cmH_2_O in the LTS-D group required inflation volumes of 67 ± 13 and 76 ± 6 ml, respectively. When the size four LTS-D was inflated with 80 ml of air as indicated on the colour-coded syringe intended for emergency use, the cuff pressure markedly exceeded the manometer scale’s maximum of 110 cmH_2_O in all cases.

### Success rates and insertion times

The overall insertion success rate was significantly lower for the LTS-D than for the other two SGA (Chi-square, *p* = 0.014; Figure 1). Insertion times did not differ significantly (i-gel™ 10 ± 5 sec; LMA-S 11 ± 9 sec; LTS-D 14 ± 10 sec; *p* = 0.173).

Three patients in the LTS-D group did not complete the entire study protocol due to complications during insertion. Despite absent response to a forced jaw thrust one patient developed severe laryngospasm immediately after insertion and required muscle relaxation and endotracheal intubation. One patient suffered a clinically relevant aspiration of gastric contents necessitating postoperative admission to the intensive care unit, and one had a suspected regurgitation of gastric contents. These were graded as insertion failures (see also Figure 1).

**Figure 3:**
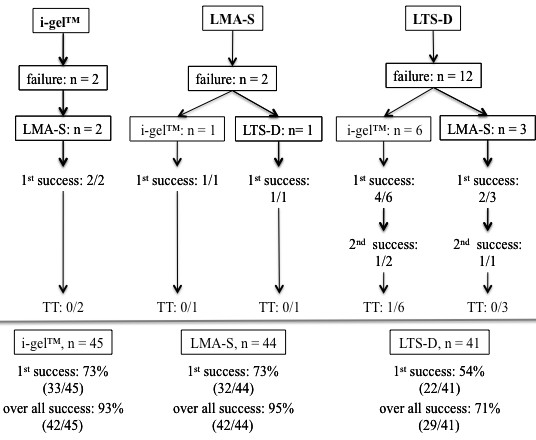
**Flow chart indicating the number of failures and the supraglottic airway device used as the secondary device, including the corresponding success rates.** 1st or 2nd success = insertion success rate for the first or the second insertion attempt. i-gel™; LMA-S = LMA-Supreme™; LTS-D = Laryngeal Tube Suction-D; TT = tracheal tube. Below the horizontal line is the summary for each device (intention-to-treat plus rescue device).

Figure 3 shows the success rates of the alternative SGA that had to be used when insertion of the initially randomized SGA failed. Analysing the primary endpoints it made no difference whether a SGA had been inserted as the primary or as the backup device. Adding randomised and alternative devices gave a total number of insertions of 45 i-gel™ (38 randomised + 7 alternative), 44 LMA-S (39 + 5), and 41 LTS-D (40 + 1) (Figure 3).

Airway pressures and C_dyn_ are shown in Table 2.

**Table 2:** Airway pressure and airway compliance

	**dPAW [cmH**_**2**_**0]**	**C**_**dyn**_**[ml/cmH**_**2**_**0]**
**i-gel™**	11.2 ± 2.8	49.9 ± 12.7
**LMS-S**	12.0 ± 0.5	43.4 ± 11.4
**LTS-D**	13.2 ± 4.3*	38.0 ± 13.4*

dPAW = delta pressure airway (=PAW_peak_ - PEEP), C_dyn_ = dynamic airway compliance. LMA-S = LMA-Supreme™; LTS-D = Laryngeal Tube Suction-D. * *p* <0.05.

### Fibreoptic assessment

**Figure 4:**
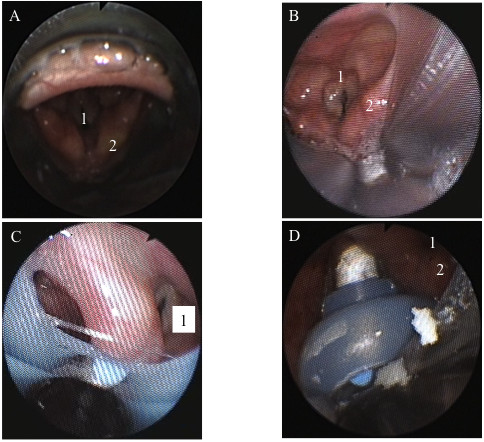
**Examples of fibrescopic images. A**) i-gel™, note that the epiglottis does not rest on the epiglottis rest outside the mask bowl; **B**) LMA-Supreme™, note the narrowing of the vocal cords; **C** and **D**) Example of awkward position in situ of the Laryngeal Tube Suction-D, ventilation was possible in both examples. 1 = glottic inlet, 2 = arytenoids.

All successfully inserted SGAs were assessed by fibrescope. The vocal cords and epiglottis were visible in all patients with an i-gel™ or a LMA-S, which corresponds to grade 2. In 40 of the 42 patients with an i-gel™ (95%), the epiglottis was not on the intended epiglottis rest as described in the user’s manual but was caught in the bowl of the mask (Figure 4A). In one of 42 patients with an LMA-S, the epiglottis was folded down. Airway obstruction was not observed in any of these patients.

Glottic narrowing that was not due to laryngeal distortion or laryngospasm was seen in three of the 42 patients (7%) with an LMA-S (see also Figure 4B).

The concept and construction of the LTS-D is different from that of the i-gel™ and the LMA-S, which makes it difficult to apply the fibreoptic scoring system. However, in 21 of 29 patients (72%) we were able to visualise laryngeal structures through one of the LTS-D’s ventilatory openings to varying degrees (grades 1 to 3). In eight of the 29 (28%) patients, no laryngeal structures were visible but we were able to ventilate the patients’ lungs nonetheless.

### Airway morbidity

The overall incidence of airway morbidity for the intention-to-treat groups was low. No statistical intergroup difference was seen regarding hoarseness. There was significantly more sore throat and dysphagia associated with the LTS-D than with the other two devices (Table 3). No difference was found between the latter. Traces of blood were found more often on the LTS-D (see also Table 4). One of the five patients with an i-gel™ with blood on the device described a slight sore throat one hour after surgery. Two patients with a blood-stained LMA-S complained of a slight sore throat and dysphagia, and one complained of hoarseness one hour after surgery. All but one of the patients with a blood-stained LTS-D complained of airway morbidity.

**Table 3:** Incidence of airway morbidity

	**sore throat**		**hoarseness**		**dysphagia**	
	**% of patients with none /mild /severe**		**% of patients with none / mild / severe**		**% of patients with none / mild / severe**	
	***1h***	***1d***	***1h***	***1d***	***1h***	***1d***
**i-gel**™	72 / 14 / 14	88 / 6 / 6	86 / 14 / 0	97 / 3 / 0	83 / 14/ 3	100 / 0 / 0
**LMA-S**	84 / 16 / 0	95 / 5 / 0	92 / 5 / 3	98 / 2 / 0	95 / 2.5 / 2.5	95 / 5 / 0
**LTS-D**	29 / 50 / 21	79 / 17 / 4	82 / 11 / 7	93 / 7 / 0	54 / 32 / 14	82 / 14 / 4
	***p*<0.001**	***p*<0.005**	***p*** **= 0.75**	***p*** **= 0.64**	***p*<0.001**	***p*<0.005**

LMA-S = LMA-Supreme™; LTS-D = Laryngeal Tube Suction-D. 1h = one hour after surgery; 1d = one day after surgery.

**Table 4:** Blood on the devices of the intention-to-treat group

	**all insertions**		**successful insertions**		**failures*without*blood**
	**blood inside**	**blood outside**	**blood inside**	**blood outside**	
**i-gel™**	8% (n = 3)	13% (n = 5)	8% (3 of 36)	14% (5 of 36)	2 of 2 (100%)
**LMS-S**	8% (n = 3)	13% (n = 5)	3% (1 of 37)	8% (3 of 37)	0 of 2 (0%)
**LTS-D**	20% (n = 8)	37.5% (n = 15)	14% (4 of 28)	35% (10 of 28)	7 of 12 (58%)
***p-*value**	= 0.132	= 0.006	= 0.216	= 0.01	

LMA-S = LMA-Supreme™; LTS-D = Laryngeal Tube Suction-D.

## Discussion

In this study we compared three different SGA, i-gel™, LMA-S, and LTS-D in surgical patients with regard to leak pressure, insertion success rate and adverse effects.

### Leak pressure and cuff pressure

Leak pressures did not differ between the devices when the inflatable devices had a cuff pressure of 60 cmH_2_O. The LP determined for the i-gel™ and the LMA-S are similar to those described previously [3,6,7], but we found a LP for the LTS-D, which is at the lower end of the range of 25 to 33 cmH_2_O described in other studies [8,16]. This might be because we measured LP at a cuff pressure at 60 cmH_2_O as recommended by the manufacturer for elective patients [18] and not at the presumably higher pressures used in other studies. The mean inflation volume required for a cuff pressure of 60 cmH_2_O was 57.9 ml, which is markedly less than that used in other studies, so that even though the actual cuff pressures in these studies were not reported, one can conclude they were higher than 60 cmH_2_O [2,16]. For emergency situations the manufacturer, of course, prefers a fail-safe pragmatic approach and provides a colour-coded 80 ml syringe that produces cuff pressures of more than 110 cmH_2_O.

Three patients were excluded from analysis because size selection of the device was not according to the prescribed weight-based guideline, even though it would have been correct according to the gender-based guideline [19]. A recalculation of LP and success rates using the data of these three patients showed that their exclusion did not affect the results, which were essentially the same as those presented in the results section (LP: i-gel™ 25.7 ± 5.7 cmH_2_0; LMA-S 26.5 ± 5.1 cmH_2_0; LTS-D 25.7 ± 3.9 cmH_2_0, *p* = 0.667; insertion success rate: *p* = 0.002).

LP correlated positively with cuff pressure, which confirms recently published data for the LMA-S [20]. This is clinically relevant, since there is less postoperative airway morbidity at lower cuff pressures [21], and the incidence and severity of sore throat is markedly reduced at cuff pressures below 40 cmH_2_O [22]. This may be due to a lower mucosal pressure as discussed below. The decrease in LP was more pronounced with the LTS-D, so that the LMA-S had higher leak pressures at cuff pressures below 50 cmH_2_0 (Figure 2).

Leak pressure of an SGA depends on a tight contact of the cuff with the surrounding tissues [23]. If the radial pressure of the cuff on the mucosa is above the mucosal perfusion pressure it will obviously cause tissue ischemia, which can contribute to airway morbidity. This radial pressure is a non-linear function of cuff pressure and depends also on the elasticity and dimensions of the cuff and the compliance of the surrounding tissue. It probably cannot be calculated directly from cuff pressure but must be determined empirically. However, Ulrich-Pur et al. concluded that increasing cuff volumes are directly transferred to increasing mucosal pressures [24] and one study with the modified cuffed oropharyngeal airway in patients did demonstrate a significant, direct correlation between cuff pressure and mucosal pressure [25].

Studies have shown that mucosal perfusion remains normal as long as the maximum mucosal pressure is lower than 32 cmH_2_O, but that perfusion is slightly reduced at mean pressures of 34 cmH_2_O with a pressure range of 4 to 65 [25]. A recent study of the i-gel™ and the LMA-S with a cuff pressure of 60 cmH_2_O in surgical patients found that maximal mucosal pressures were lower than 38 cmH_2_O and that there was no difference between the two devices [26]. Other laryngeal mask airways and laryngeal tubes have been studied in cadavers with conflicting results [24,27,28], however, the recorded mucosal pressures were generally below the ischemia threshold.

### Success rates

The success rates with the i-gel™ and the LMA-S are similar to published data [3,7]. The insertion success rate with the LTS-D in this study was significantly lower than for the other two devices but still lay within the range of published data [2,16,29-32]. Schalk et al. [32] had high success rates with the LTS II and LTS-D, while Kette et al. [33] and Heuer at al. [34] described successful ventilation in only 75-80% of the cases. There may be methodological reasons for the low insertion success rates in our study. First of all, we limited the number of insertion attempts to two and set somewhat higher criteria for a successful insertion, i.e. absence of any air leak at sufficient tidal volumes in addition to simply being able to ventilate. Another possible factor might be that the two investigators had less experience inserting the LTS-D than they had with devices similar to the i-gel™ and the LMA-S (classical laryngeal mask airway and PLMA). On the other hand, it is claimed that the laryngeal tube is easy to insert, even for persons with little experience [32]. A third factor might have been inadequate anaesthesia. None of our patients responded to the forced jaw thrust, generally considered a reliable sign that anaesthesia is deep enough to insert an LMA [9,10], and several studies have shown that a similar depth of anaesthesia was required for laryngeal tube and laryngeal mask airway insertion [35-37]. One study even found that insertion conditions were better for the laryngeal tube at the same depth of anaesthesia [35]. On the other hand, there is data showing that laryngeal tube insertion requires a deeper level of anaesthesia [38], and in view of the fact that some of our patients responded to the insertion attempts, the level of anaesthesia might have been insufficient.

### Airway position, compliance and morbidity

The employed scoring system gave identical ratings for the position of the i-gel™ and the LMA-S. There was no airway obstruction, even when the epiglottis was kinked or otherwise not in the correct position.

The lower scores for the LTS-D confirm previous findings. Bortone et al. compared the laryngeal tube with a classic LMA in children and found that no laryngeal structures were identifiable in six of eleven children [39]. Kim et al. demonstrated that the view of the laryngeal structures during the fibreoptic assessments of the LTS-II depended significantly on the position of the head [40]. Mihai et al. were able to visualize the glottis only in 51% of the cases with the LTS-II [30]. In our study, ventilation was possible even though the correct position of the device could not be confirmed (e.g. Figure 4C and D).

Dynamic airway compliance was lowest for the LTS-D. This is in accordance with the data of Gaitini et al. who reported that a significantly higher peak airway pressure was required to achieve comparable tidal volumes [41] and with those of Cook et al., who reported that ventilation efficacy was higher with the PLMA than with the LTS [42]. Based on the results of our fibreoptic evaluations, one could postulate that the lower airway compliance may be partially caused by pharyngeal tissue (epiglottis, pharyngeal wall) obstructing the airway openings.

We observed a glottic narrowing not attributable to insufficient anaesthesia in 7% of the patients with an LMA-S. This is in accordance with previous results that described an incidence of approximately 10% [7]. Neither in this, nor in our previous study [7] did glottic narrowing necessitate removal of the LMA-S. However, glottic narrowing is an unintended clinical occurrence not fully understood as the anatomical position of the LMA-S appears to be correct and, importantly, the condition cannot be reversed by deepening the anaesthesia or by administering muscle relaxants. It is our experience that the only option to facilitate glottic opening is to remove and to reinsert the device.

The greater incidence of blood on the devices was significant for the LTS-D and could possibly also be so for the i-gel™. The latter result would confirm that of Eschertzhuber et al. [26]. Although our study was neither designed nor powered to detect a relationship between blood on the devices and airway morbidity, it appears that there might be an association between mucosal injury and airway morbidity with the LTS-D.

### Limitations

The observer who measured the insertion times was not blinded to the type of SGA being used. The investigators inserting the devices had less experience with the LTS-D than with devices similar to the other two. The cuff of the LTS-D but not that of the LMA-S was briefly (<60 seconds) inflated to 100 cmH_2_O for inflation volume and LP measurements. The study was not powered to draw conclusions on small differences in airway morbidity.

## Conclusions

All devices were found to be suitable for ventilating the patients’ lungs during elective surgery. Leak pressures did not differ, but airway morbidity was more pronounced with the LTS-D.

## Competing interests

This study was funded by departmental resources only. The corresponding author declares that he had sole and complete control over collection of data, data analysis, interpretation of the results and content of the manuscript. He was not influenced by any company.

## Authors’ contributions

SGR designed and conducted the study, analyzed the data, and wrote the manuscript. SC conducted the study, analyzed the data, and drafted the manuscript. TG conducted the study. CE, AB and MB helped designing the study and writing the manuscript. TAC drafted the manuscript. MS analyzed the data and helped to write the manuscript. All authors read and approved the final manuscript.

## Pre-publication history

The pre-publication history for this paper can be accessed here:

http://www.biomedcentral.com/1471-2253/12/18/prepub
